# Research hotspots and trends of urology education in China and worldwide over the past decade: A Bibliometric Comparative Analysis

**DOI:** 10.1097/MD.0000000000050085

**Published:** 2026-08-21

**Authors:** Yaxiong Tang, Yun Tan, Gang Chen, Cheng Ren

**Affiliations:** a Department of Urology, The First People’s Hospital of Neijiang, Neijiang, Sichuan, China; b Health Management Center, The First People’s Hospital of Neijiang, Neijiang, Sichuan, China; c Department of Urology, The First Affiliated Hospital of Chongqing Medical University, Chongqing, China; d Department of Urology, The First People’s Hospital of Neijiang, Neijiang, Sichuan, China.

**Keywords:** bibliometrics, clinical competence, internship and residency, medical education, urology

## Abstract

**Background:**

To systematically compare the research landscape, thematic hotspots, and evolutionary trajectories of urology education in China and globally between 2015 and 2024, and provide evidence-based insights for optimizing urology residency training and clinical teaching reform.

**Methods:**

We searched for urology education papers published between 2015 and 2024 in Chinese databases (China National Knowledge Infrastructure (CNKI), Wanfang, VIP, Chinese dissertation database, National Urology Education Symposium proceedings) and international databases (Web of Science Core Collection, PubMed, ProQuest dissertation database, American Urological Association (AUA)/European Association of Urology ) annual meeting proceedings). Bibliometric methods were applied for keyword co-occurrence, cluster analysis, and burst detection using VOSviewer 1.6.19, CiteSpace 6.4.R1, and Bibliometrix 4.1.0. The validated Medical Education Research Study Quality Instrument (MERSQI) was used for quality control. Literature screening followed an adapted Preferred Reporting Items for Systematic Reviews and Meta-Analyses (PRISMA) 2020 statement.

**Results:**

We identified a total of 8217 records from all databases. After screening and quality evaluation, 1563 studies were finally included in the bibliometric analysis (1137 from China, 426 from other countries). Annual publication volume showed steady growth in both regions (average annual growth: 6.2% in China, 6.6% globally). Four core clusters were identified in Chinese studies: teaching methodology innovation, technology-assisted instruction, standardized residency training optimization, and professionalism cultivation, reflecting a practice-oriented problem-solving paradigm. Four clusters were identified in international studies: interprofessional education (IPE), continuing professional development. simulation training system construction, and nontechnical skills training, reflecting a theory-driven system-building paradigm. The mean MERSQI score was 6.2 for Chinese studies and 9.7 for international studies, indicating a substantial gap in methodological rigor.

**Conclusions:**

Urology education research has expanded rapidly worldwide over the past decade, with distinct developmental paths between China and Western countries. Future efforts in China should prioritize localized adaptation of educational technologies, construction of standardized specialty education systems, and improvement of research methodology to advance high-quality urology workforce development.

## 1. Introduction

Urology demands both a deep theoretical foundation and advanced technical skills, making it 1 of the most challenging surgical specialties. The quality of urology training directly determines clinicians’ clinical proficiency of practicing urologists and impacts patient safety and healthcare outcomes.^[1]^ Minimally invasive surgical techniques have gained widespread use over the past decade. Virtual reality (VR) and artificial intelligence(AI)-assisted training tools are increasingly integrated into clinical teaching. Moreover, Population aging also drives medical schools to shift from single offline courses to hybrid online-offline training models.

Bibliometric analysis enables quantitative mapping of publication characteristics, keyword networks, and institutional collaboration patterns, providing objective insights into research hotspots and disciplinary evolution to inform evidence-based educational reform.^[2]^ Currently, urology education research in China faces persistent challenges including uneven resource distribution, underdeveloped evaluation frameworks, and regional disparities in training quality. Most existing studies are single-center evaluations of individual teaching methods, and most published bibliometric studies are limited to journal articles, excluding frontier outputs such as dissertations and conference proceedings.

Globally, bibliometric studies have mapped trends in surgical simulation training^[3]^ and its associated learning curves,^[4]^, and a 30-year overview of urology education research exists, but few have conducted in-depth cross-national comparative analysis.^[5]^ This study presents a 10-year bibliometric comparison of urology education research in China and worldwide, incorporating dissertations and conference literature with standardized quality assessment via the Medical Education Research Study Quality Instrument (MERSQI) scale. We aim to delineate differences in research priorities and developmental pathways, and provide actionable references for optimizing the urology specialty training system in China.

## 2. Methods

### 2.1. Study design

This is a retrospective cross-national bibliometric comparative study designed to systematically compare urology education research between China and global regions. We followed the Preferred Reporting Items for Systematic Reviews and Meta-Analyses (PRISMA) 2020 reporting framework and adjusted its criteria to fit bibliometric research. We deleted all items designed for clinical trials but fully retained the 4-step screening workflow: identification, preliminary screening, full-text eligibility assessment, and final inclusion. The inter-rater reliability test (Cohen’s kappa = 0.87) demonstrated excellent screening consistency between 2 independent researchers during the literature evaluation process. No protocol was registered because this analysis used only publicly available data.

### 2.2. Data sources and search strategy

We established 2 independent retrieval libraries for Chinese and global literature, respectively:

Chinese literature databases: China National Knowledge Infrastructure(CNKI), Wanfang Data, Chongqing VIP Chinese Science and Technology Periodical Database, Chinese Doctoral Dissertations and Master’s Theses Full-text Database, and the proceedings of the National Urology Education Symposium. International literature databases: Web of Science Core Collection, PubMed, ProQuest Dissertation Database, and official proceedings of the AUA and annual meetings. No supplementary manual gray literature search was performed.

The retrieval time frame was January 1, 2015 to December 31, 2024. A combination of Medical Subject Headings (MeSH) and free-text terms was applied:

- **English retrieval formula (combining MeSH major terms + free text words):** (Urology[MeSH Terms] OR Urological Surgery[Title/Abstract]) AND (Medical Education[MeSH Terms] OR Teaching[Title/Abstract] OR Training[Title/Abstract] OR Clinical Education[Title/Abstract] OR Residency Training[MeSH Terms]).
- **Chinese retrieval formula:** (泌尿外科) AND (教学 OR 教育 OR 培训 OR 临床教学 OR 规范化培训).

### 2.3. Inclusion and exclusion criteria

#### 2.3.1. Inclusion criteria

Research subjects focused on urology specialty teaching, including classroom theory teaching, clinical skill training, training evaluation system and urologist competency cultivation; Document types: peer-reviewed journal articles, doctoral/master’s dissertations, and formal academic conference papers; Languages restriction: Chinese or English full-text literature; Complete research dataset and clear, reportable research outcomes; Methodological quality score assessed by MERSQI ≥ 4 (moderate and above quality).

#### 2.3.1. Exclusion criteria

Pure narrative reviews and meta-analyses (only cited in Discussion for theoretical background, not incorporated into quantitative bibliometric dataset to avoid repeated counting); Case reports, letters to the editor, editorials comments and other non-original research short communications; Literature irrelevant to the theme of urology education; Duplicate published works; only the most complete latest version was reserved; Low-quality literature with MERSQI score ≤3.

### 2.4. Literature screening and data extraction

Two independent researchers completed literature screening and data extraction in parallel without mutual communication to reduce subjective bias. All inconsistent screening results were resolved through group discussion with a third senior urology education researcher to reduce subjective bias. EndNote software X9 was used for centralized duplicate removal. The screening workflow was divided into 2 phases: preliminary screening by titles and abstracts, and secondary full-text eligibility screening. Unified standardized variables were extracted from qualified literature, including publication year, database source, author affiliation, regional distribution, hospital grade, author keywords and core research themes. Keyword unification adopted a 2-step calibration strategy: automatic high-frequency keyword extraction via CiteSpace, followed by manual synonym consolidation and term standardization by 2 researchers according to urology education disciplinary consensus. Cohen’s kappa statistic was calculated to evaluate inter-rater screening consistency.

### 2.5. Quality assessment

We adopted the complete MERSQI scale to evaluate the methodological quality of all included papers. MERSQI full name: MERSQI, a verified evaluation tool with 6 independent scoring dimensions: study design, sampling method, data type, tool validity, statistical analysis, and research outcome. The total score ranges from 0 to 18: ≥11.0 = high-quality research; 4.0 to 10.0 = moderate quality; ≤3.0 = low-quality. Two raters scored separately, and disputes were resolved through group discussion. Inter-rater reliability was calculated using Cohen’s kappa, with κ = 0.87, showing excellent agreement among 3 independent raters.^[6]^

### 2.6. Statistical and bibliometric analysis

We calculated basic descriptive data via Excel 2021. For visualization analysis, we set unified CiteSpace parameters: time slice = 1 year, TopN = 50, log-likelihood ratio (LLR) cluster labeling, Pathfinder pruning, minimum co-occurrence threshold = 5. We set TopN = 50 to retain the top 50 highest-frequency terms for each time slice, and pruning threshold = 5 to filter low-frequency nodes; these parameter settings follow widely adopted bibliometric analysis practices from classic CiteSpace methodological publications, to balance network readability and information completeness. We conducted keyword co-occurrence, cluster division and burst detection in sequence.

### 2.7. Ethical considerations

Ethical approval was not required as this study only involved secondary analysis of publicly available published data, with no human participants, animal subjects, or identifiable personal information.

## 3. Results

### 3.1. Literature screening process

**Figure 1.**
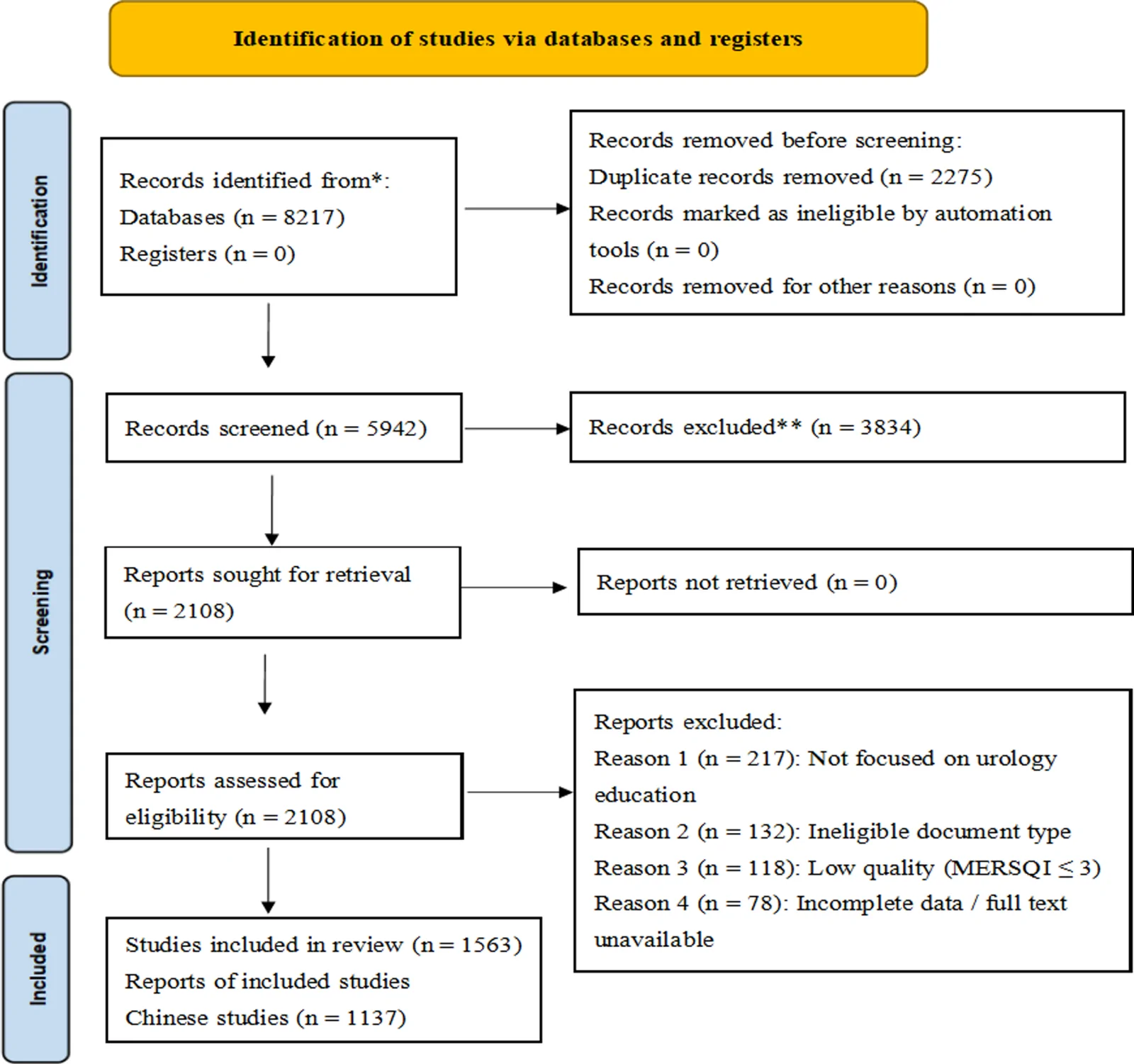
Adapted PRISMA 2020 four-stage literature screening flowchart. PRISMA = Preferred Reporting Items for Systematic.

A total of 8217 records were initially retrieved across all sources, including 5320 from Chinese databases and 2897 from international databases. No additional records were identified via manual gray literature searches or other supplementary sources. The full 4-phase workflow of identification, screening, eligibility assessment, and final inclusion is summarized in an adapted PRISMA 2020 flow diagram (Fig. 1).

After duplicate removal using EndNote X9, 5942 unique records remained for title and abstract screening. Of these, 3834 records were excluded due to irrelevant research topics, ineligible document types, or residual duplicate publications, leaving 2108 records that advanced to full-text eligibility review.

During full-text assessment, 545 records were excluded for predefined reasons: 217 studies were not focused on urology education, 132 did not meet the document type inclusion criteria, 118 were rated as low methodological quality (MERSQI score ≤3), and 78 had incomplete datasets or unavailable full texts.

Ultimately, 1563 valid studies met all inclusion criteria and were included in the final bibliometric analysis, comprising 1137 studies from China and 426 from other countries. The Cohen’s kappa value for inter-rater screening agreement was 0.87, indicating substantial inter-rater reliability.

### 3.2. Basic characteristics of included studies

#### 3.2.1. Annual publication trends

**Figure 2.**
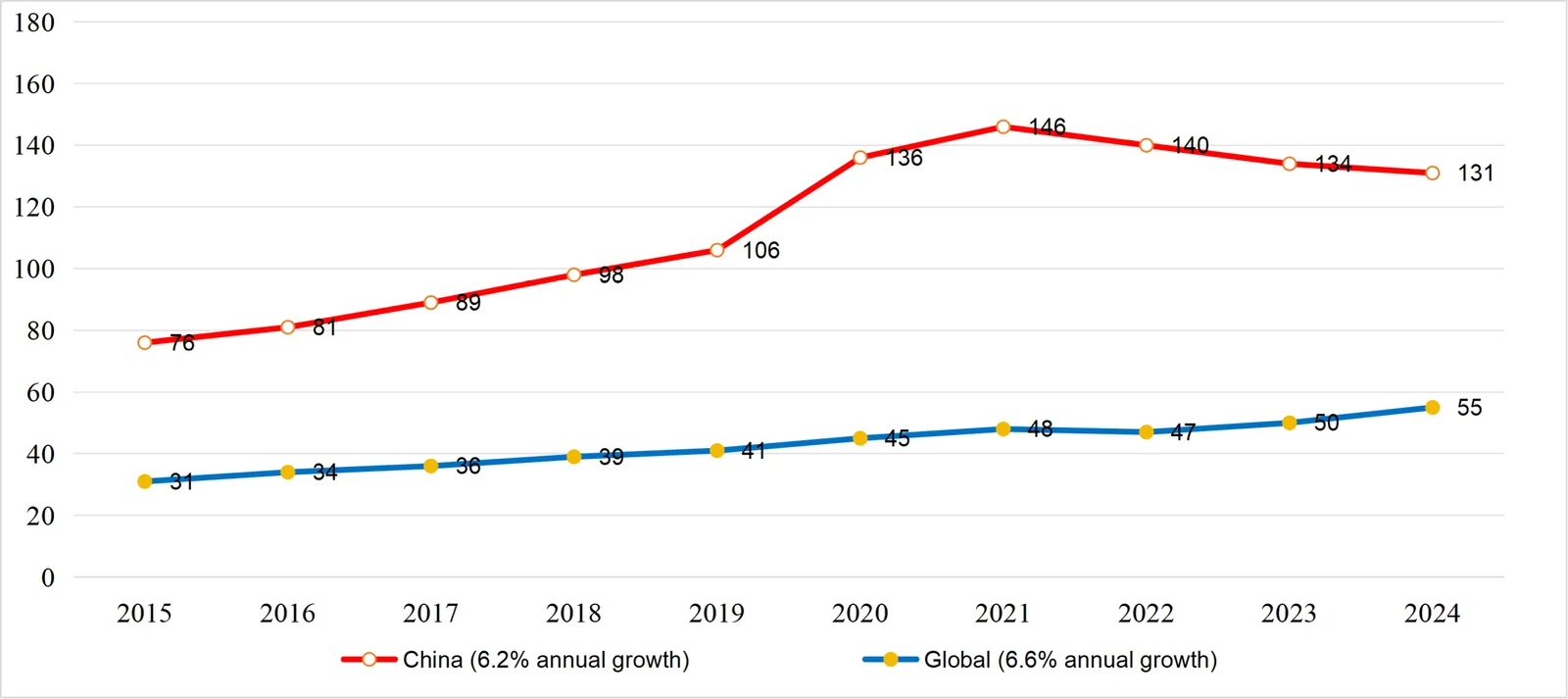
Annual publication trends of Chinese and global urology education research (2015–2024).

Annual publication output maintained steady growth in both China and global regions over the decade (Figure 2, Table 1). Detailed annual publication quantities and average annual growth rates from 2015 to 2024 are summarized in Table 1. A prominent growth surge occurred during 2020 to 2022. The COVID pandemic restricted offline clinical training, so many scholars turned to virtual online teaching research. Robotic surgery standardized training had already developed into a mature research cluster in global literature by 2018.

**Table 1:** Annual publication output of urology education research (2015–2024).

Yr	Chinese studies (n = 1137)	Global studies (n = 426)
2015	76	31
2016	81	34
2017	89	36
2018	98	39
2019	106	41
2020	136	45
2021	146	48
2022	140	47
2023	134	50
2024	131	55
**Average annual growth rate**	**6.2%**	**6.6%**

Values are presented as number of publications. Percentages for growth rates are rounded to 1 decimal place.

#### 3.2.2. Publication types and methodological quality

Distinct structural differences existed in document types between Chinese and global literature samples, with full composition proportions presented in Table 2. Chinese research relies more on journal publications, while international datasets contain a slightly higher share of conference manuscripts. A larger proportion of global studies were published in high-impact JCR Q1/Q2 journals compared with Chinese core publications.

**Table 2:** Characteristics of included literature by region.

Category	Chinese studies (n = 1137)	Global studies (n = 426)
Document types		
Journal articles	1052 (92.5%)	376 (88.3%)
Dissertations	68 (6.0%)	32 (7.5%)
Conference papers	17 (1.5%)	18 (4.2%)
Top productive institutions		
1st	Shengjing Hospital (18)	UCSF (22)
2nd	Anhui Medical University (14)	Harvard Medical School (19)
3rd	PLA General Hospital (12)	University of Sydney (16)
Geographic distribution		
China eastern regions	58.3%	–
North America	–	42.3%
Europe	–	35.7%
Oceania	–	15.0%
Primary care focus	–	31.2%
Cross-regional collaboration	11.0%	36.0%

All percentages are rounded to 1 decimal place. Institution names are listed with publication counts in parentheses.

PLA = People’s Liberation Army.

Notable gaps in methodological quality were observed between the two cohorts. The average MERSQI score reached 9.7 for global literature versus 6.2 for Chinese studies, and the proportion of high-quality studies with MERSQI≥11 was far higher in global publications (41.5% vs 8.2%). Most Chinese studies only adopt simple pre-post comparative designs and lack rigorous control groups.

#### 3.2.3. Institutional and geographic distribution

Urology education research from Chinese institutions is highly concentrated in tertiary hospitals in eastern China, with extremely low cross-regional collaborative output, showing fragmented regional research resources.

Global research forms a stable transnational cooperation network dominated by North American and European top medical schools, with much higher cross-border collaboration rates than China. A large share of international literature targets community primary care and continuous professional development, which remains an understudied area in Chinese urology education research.

### 3.3. Keyword co-occurrence and cluster analysis

#### 3.3.1. Thematic hotspots in Chinese research

**Figure 3.**
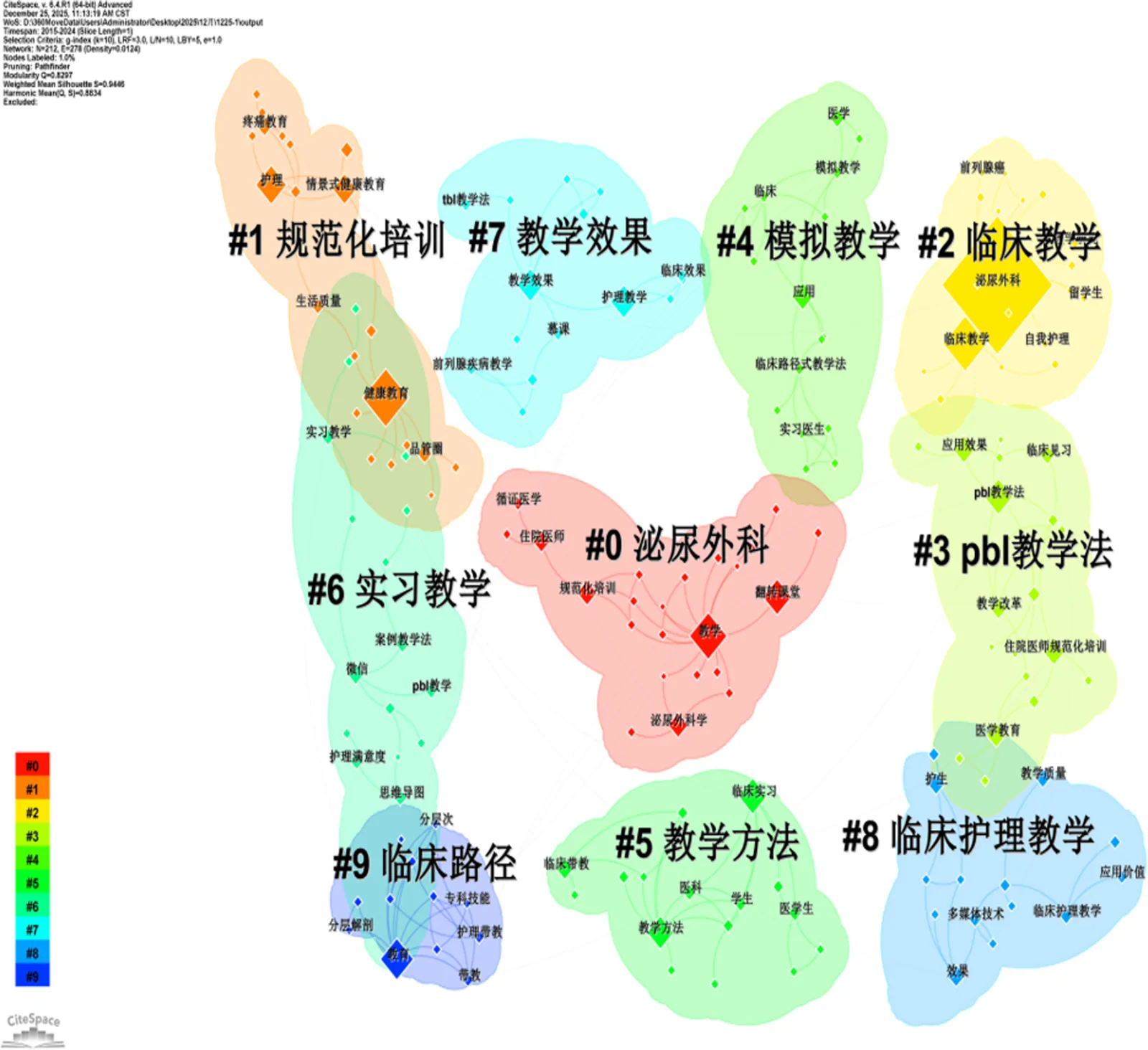
Keyword clustering map of Chinese urology education research.

A total of 283 unique keywords were extracted from Chinese studies. All keyword network visualizations and cluster partitioning were processed via CiteSpace 6.4.R1, with unified analytical parameters: minimum keyword co-occurrence threshold set to five, LLR algorithm adopted for cluster label extraction, and Pathfinder pruning applied to simplify redundant network connections. High-frequency terms included problem-based learning, case-based learning (CBL), standardized residency training, VR, laparoscopic teaching, and 3D printing. Four core thematic clusters were identified (Fig. 3): innovative integration of teaching methodologies; technology-assisted clinical instruction; optimization of standardized residency training; professionalism and humanistic communication cultivation.

Detailed connotation of each Chinese cluster is interpreted as follows: Cluster 1 focuses on the combination and localized improvement of classic classroom teaching modes represented by problem-based learning and CBL; Cluster 2 centers on the auxiliary application of digital teaching equipment such as VR and 3D printing in surgical skill training; Cluster 3 takes national resident standardized training policy as the core, exploring practical optimization paths for clinical training management; Cluster 4 concentrates on soft skill cultivation including doctor-patient communication, medical ethics and humanistic literacy.

The keyword network presents a layered framework linking various teaching instruments. Its core node is standardized residency training (centrality = 0.42) as the sole core node connecting all 4 clusters. Betweenness centrality quantifies how frequently a node acts as the shortest path bridge between other network nodes; higher values indicate the node has stronger bridging influence in the keyword co-occurrence network. The overall network density was 0.21, with a modularity Q value of 0.32 and average silhouette coefficient of 0.71. While the modularity Q falls slightly below the conventional 0.40 threshold commonly used to indicate strong cluster separation, the silhouette coefficient of 0.71-well above the 0.50 benchmark: confirms that the within-cluster coherence is good and the cluster assignments are reliable for comparative interpretation. The network showed a pattern of isolated single-point innovation: teaching method research was rarely integrated with technical tools, and technology applications were largely limited to standalone efficacy verification rather than embedded within formal training systems. Burst detection identified “VR” and “professionalism” as strong burst keywords emerging in 2020 (burst strength: 8.2 and 6.7, respectively), persisting through 2024. The high burst intensity of VR keywords after 2020 reflects the rapid growth of digital simulation research, caused by suspended offline clinical training amid the COVID-19 pandemic.

#### 3.3.2. Thematic hotspots in international research

**Figure 4.**
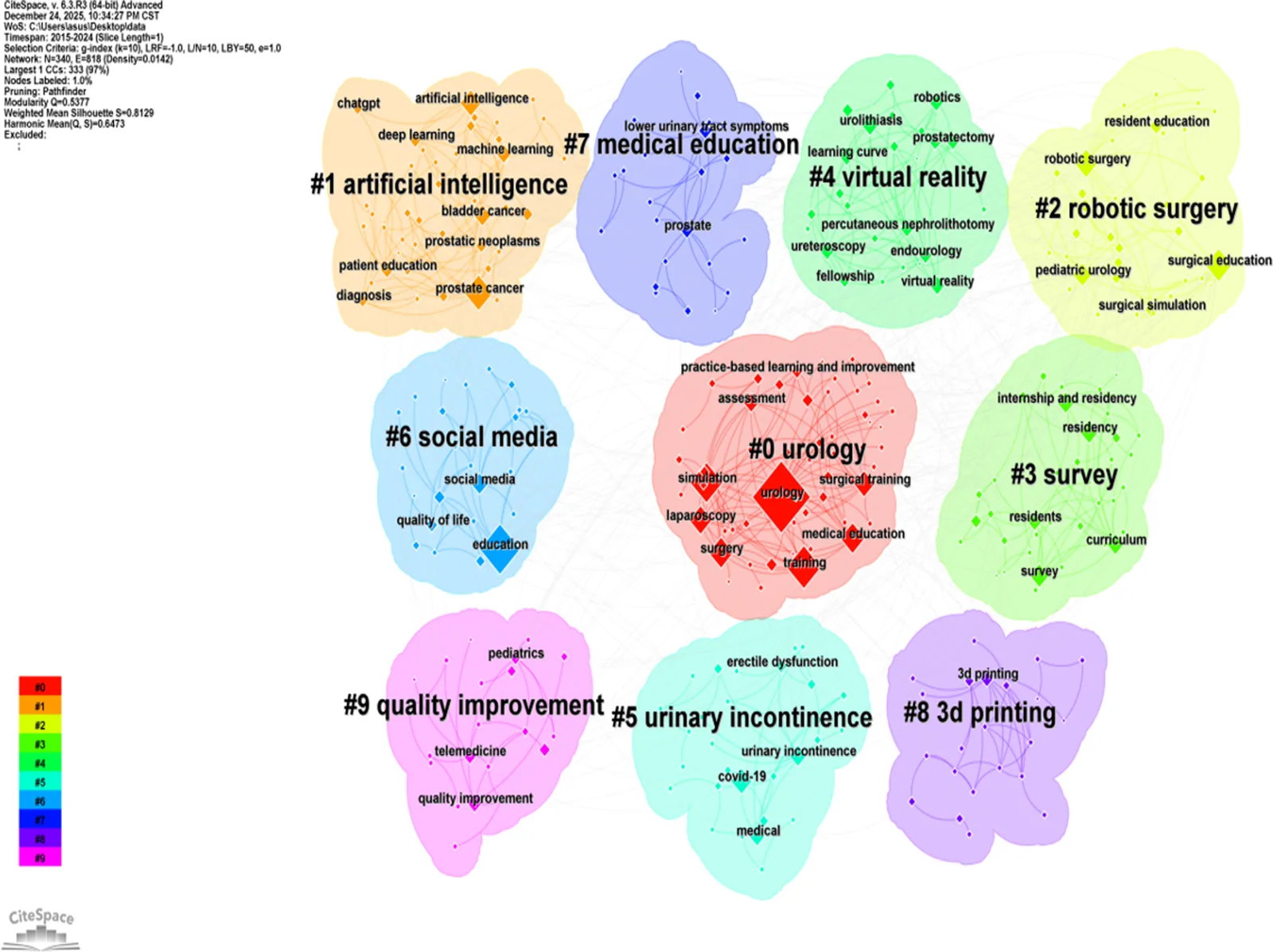
Keyword clustering map of international urology education research.

A total of 207 unique keywords were extracted from international studies. Consistent CiteSpace parameter settings were used for international keyword clustering, including a minimum co-occurrence threshold of 5, LLR labeling, and Pathfinder network pruning to ensure comparability between Chinese and global network results.High-frequency terms included interprofessional education (IPE), continuing professional development simulation training, robotic surgery training, and primary care. Four core thematic clusters were identified (Fig. 4): IPE framework; continuing professional development systems; simulation training standardization; nontechnical skills training.

Each international cluster carries systematic theoretical orientation: Cluster 1 constructs multi-disciplinary collaborative training models around interprofessional teamwork; Cluster 2 establishes lifelong hierarchical training systems for practicing urologists after residency; Cluster 3 forms unified standard operating specifications for high-fidelity surgical simulation; Cluster 4 builds independent curriculum systems for communication, crisis management and other nontechnical abilities.

The international clusters formed an integrated closed-loop structure anchored to competency-based medical education (CBME). The betweenness centrality of “CBME” reached 0.57, serving as the unifying theoretical framework across all clusters. Inter-cluster connectivity was significantly higher than in the Chinese network, with a network density of 0.38, modularity *Q* of 0.35 and silhouette coefficient of 0.76. The silhouette coefficient of 0.76 indicates excellent within-cluster coherence, and while the modularity *Q* is slightly below the conventional 0.40 threshold, the network visualization and the high silhouette score both support the validity of the 4-cluster structure. This structural difference indicates that international urology education research forms a mutually supportive systematic research system, while Chinese research remains scattered, independent single-point explorations. Burst detection showed that “AI” and “nontechnical skills” became strong burst keywords from 2021 (burst strength: 7.9 and 6.3, respectively), representing the current international research frontier. The sustained high burst strength of AI reveals that intelligent automatic skill assessment has become the core innovative direction of global surgical education research in recent years. This observation is consistent with broader bibliometric findings, which confirm that the rapid expansion of AI research in medical education over the past decade has seen surgery and simulation-based training consistently emerging as core thematic hotspots.^[7]^

#### 3.3.3. Comparative evolution of research hotspots

The evolution of urology education research fell into 3 distinct stages, with thematic priorities diverging markedly between China and the rest of the world (Table 3). Shifts in Chinese research hotspots were driven primarily by policy initiatives and expanding technology application scenarios, while international evolution was guided primarily by educational theory development and advances in clinical specialty technology.

**Table 3:** Comparative evolution of urology education research hotspots in China and worldwide.

Time period	Chinese research hotspots	International research hotspots
2015–2017	Optimization of traditional teaching methods (PBL, CBL); foundational development of urology standardized residency training	Establishment of interprofessional education systems; standardization of simulation training protocols
2018–2020	Early adoption of technology-assisted teaching (VR, 3D printing); expansion of teaching resources for primary care settings	Refinement of hierarchical robotic surgery training systems; optimization of CPD evaluation mechanisms
2021–2024	Integrated application of multi-modal teaching methods; deeper integration of technology into curricula; emphasis on professionalism and communication skills	AI-enabled skills assessment and personalized training; systematic nontechnical skills curricula; global multicenter educational collaboration

## 4. Discussion

### 4.1. Key findings and interpretation

Our 10-year bibliometric comparison reveals steady global growth in urology education research, yet the developmental paths of China and Western countries have diverged in fundamental ways. Chinese urology education studies center on practical clinical challenges. Frontline clinician-educators initiate most of these studies, responding to real-world training needs, insufficient resident operative proficiency or limited teaching resources in primary care settings. Research objectives focus on resolving immediate practical problems, following a pattern of pilot testing, efficacy verification, and experience summarization.^[8]^ While this enables rapid response to local training needs, it also produces fragmented findings, often lacking overarching theoretical framing and long-term systemic planning.

In contrast, international research operates within a theory-driven, system-building paradigm underpinned by a mature medical education as an independent academic discipline. Most studies are guided by CBME principles, with the goal of refining training standards and producing generalizable, scalable curricula.^[1,9]^ Mature specialty education accreditation frameworks provide unified evaluation standards, enabling incremental progress from isolated innovations to system-wide improvement. For instance, the European School of Urology has established a full-cycle standardized training system through the Standardisation in Surgical Education programme, covering stepwise surgical skills, nontechnical skills, and continuing professional development, serving as a paradigm of international specialty education construction.^[10]^

Second, there is a considerable difference in methodological quality. The low mean MERSQI score among Chinese studies reflects a broader lack of formal educational research training among clinical teaching faculty. Most Chinese studies employ simple pre-post designs, with few adopting rigorous randomized controlled trials or longitudinal cohort studies. Even in well-resourced tertiary hospitals, most studies remain single-center observational evaluations, limiting generalizability.^[11]^ In comparison, the methodological quality of medical education research has been systematically evaluated using validated instruments such as the MERSQI, with international studies generally demonstrating more rigorous study designs and higher-quality evidence.^[12]^

Third, the depth of technology integration differs substantially. In China, educational technologies such as VR and 3D printing remain largely at the “tool replacement” level, with research focused on verifying the efficacy of individual technologies rather than embedding them within formal training curricula. Limited industry-academia collaboration results in a disconnect between technology development and clinical teaching needs, and a shortage of locally adapted learning modules further restricts widespread adoption in primary care settings.^[13]^ Internationally, simulation training has become a mandatory component of standardized residency training, and AI assessment tools are fully integrated into systems. Evidence from systematic reviews confirms that simulation-based training in urology has sound construct, face and content validity, and can effectively transfer technical skills to clinical operative practice, providing high-level evidence for standardized simulation curricula.^[14]^ Structured, stepwise training models have been shown to improve teaching efficiency in laparoscopic procedures.^[15]^

It is worth noting that the modularity *Q* values for both the Chinese (0.32) and international (0.35) networks fell slightly below the conventional 0.40 threshold. This modest *Q* value is not uncommon in bibliometric analyses of narrowly defined research fields: such as urology education: where the total publication volume and keyword diversity are limited compared to broader biomedical domains. Importantly, the average silhouette coefficients (0.71 and 0.76, respectively) were well above the 0.50 benchmark, confirming that the cluster structures appear internally coherent and are likely meaningful for comparative analysis.

### 4.2. Implications for clinical practice and policy

These findings have direct implications for improving urology training quality and building a competent urology workforce in China, ultimately supporting better patient care and surgical safety.

Localized development and tiered implementation of educational technologies should be prioritized. Led by professional societies, collaborative initiatives involving universities, medical device enterprises, and clinical institutions should support development of low-cost, locally adapted VR/AI training platforms paired with standardized curricula and evaluation metrics. We propose a phased implementation roadmap for tiered training reform: short-term (1–2years), complete localized translation and adaptive revision of mature international CBME evaluation tools; mid-term (3–5years), establish regional shared surgical simulation training centers covering urban and grassroots medical institutions; long-term (5+ years), build a unified national evaluation system for urology specialty education to narrow cross-regional training gaps. Tiered strategies are needed: tertiary hospitals may focus on high-fidelity simulation and AI-powered assessment, while primary care settings may prioritize simplified skills modules and remote resource sharing to reduce regional disparities.

Another key priority is to establish a standardized specialty education system. Aligning with international CBME standards and adapted to China’s healthcare context, a formal urology specialty education accreditation system should be developed, creating a full-cycle closed loop of “standardized training: competency assessment: continuous professional development,” underpinned by established principles of professional competence assessment.^[16]^ IPE principles should be gradually integrated into residency curricula to strengthen trainees’ multidisciplinary care capabilities.

A third essential component is that capacity building in educational research methodology must be strengthened. Training in medical education research methods should be incorporated into specialty training programs to develop clinician-educators with both clinical expertise and research competence. A localized quality evaluation standard for urology education research should be developed, informed by the MERSQI framework. A national urology education research collaborative network could support multicenter, large-scale studies to generate higher-quality evidence.

### 4.3. Limitations

This study has several limitations. First, only Chinese and English language literature were included, which may introduce language bias and omit studies published in other languages. Publication language bias may further aggravate selection bias in cross-region comparison: most Chinese domestic studies are published in Chinese-language journals, while international research outputs are mainly distributed in English journals; such language difference may cause unbalanced literature coverage and interfere with comparative conclusions. Second, only the specified databases were searched, and some regional conference literature and gray literature were not captured, which may lead to database selection bias and miss locally distinctive studies. Third, keyword standardization may carry minor subjective bias despite manual validation. Fourth, the MERSQI scale has limited granularity for evaluating mixed-methods research, which may introduce slight subjectivity in quality grading. Fifth, this study did not conduct cost-benefit comparisons of various training modes; relevant health economic analyses can be carried out in follow-up research.

## 5. Conclusions

The past decade has witnessed rapid expansion of urology education research in both China and the rest of the world, yet the two have followed markedly different paths. Chinese research is driven by practical clinical teaching challenges, focusing on the application of teaching methods and technologies with an overall fragmented character. International research is anchored in educational theory, focusing on standardized system-building with a mature closed-loop training framework. Moving forward, building on international experience and adapted to China’s medical education context, efforts should focus on strengthening systemic specialty education construction, advancing localized adaptation of teaching technologies, and improving research methodological rigor to achieve high-quality and equitable development of urology education.

## Acknowledgments

This research received no dedicated funding from any foundation, university research project or medical society grant. We sincerely appreciate all scholars whose published urology education literature provided the raw data basis for this bibliometric analysis. We also express our gratitude to the professional researcher who offered technical guidance on bibliometric software operation and manuscript revision. Special thanks go to the journal editorial team and anonymous reviewers for their valuable comments and constructive suggestions that greatly improved the quality of this manuscript.

## Author Contributions

**Data curation:** Yun Tan, Cheng Ren.

**Formal analysis:** Yun Tan.

**Validation:** Cheng Ren.

**Validation:** Cheng Ren.

**Writing – original draft:** Gang Chen.

**Writing – review & editing:** Yaxiong Tang.

**Writing – review & editing:** Yaxiong Tang.

**Writing – review & editing:** Yaxiong Tang.
